# PTEN: an emerging target in rheumatoid arthritis?

**DOI:** 10.1186/s12964-024-01618-6

**Published:** 2024-04-26

**Authors:** Pan Zhou, Xingwen Meng, Zhimin Nie, Hua Wang, Kaijun Wang, Aihua Du, Yu Lei

**Affiliations:** 1 Chengdu Rheumatology Hospital, Chengdu, Sichuan Province China; 2 Nanjing Tongshifeng Hospital, Nanjing, Jiangsu Province China; 3 Zhengzhou Gout and Rheumatology Hospital, Zhengzhou, Henan Province China

**Keywords:** PTEN, Rheumatoid arthritis, PI3K/AKT, Fibroblast-like synoviocyte, Target

## Abstract

Phosphatase and tensin homolog deleted on chromosome 10 (*PTEN*) is a critical tumor suppressor protein that regulates various biological processes such as cell proliferation, apoptosis, and inflammatory responses by controlling the phosphatidylinositol 3-kinase (*PI3K*)/protein kinase B (*PI3K/AKT*) signaling pathway. *PTEN* plays a crucial role in the pathogenesis of rheumatoid arthritis (RA). Loss of *PTEN* may contribute to survival, proliferation, and pro-inflammatory cytokine release of fibroblast-like synoviocytes (FLS). Also, persistent *PI3K* signaling increases myeloid cells’ osteoclastic potential, enhancing localized bone destruction. Recent studies have shown that the expression of *PTEN* protein in the synovial lining of RA patients with aggressive FLS is minimal. Experimental upregulation of *PTEN* protein expression could reduce the damage caused by RA. Nonetheless, a complete comprehension of aberrant *PTEN* drives RA progression and its interactions with other crucial molecules remains elusive. This review is dedicated to promoting a thorough understanding of the signaling mechanisms of aberrant *PTEN* in RA and aims to furnish pertinent theoretical support for forthcoming endeavors in both basic and clinical research within this domain.

## Introduction

Rheumatoid arthritis (RA) is a chronic autoimmune disorder that targets joints and is characterized by persistent synovial inflammation, proliferation of synovial tissue, synovial lichen planus, and subsequent damage to nearby articular cartilage and bone [1]. It impacts around 1% of the world’s population [2]. Typically diagnosed between the ages of 40 and 50, this condition is more common in females, with a three to five times greater occurrence rate compared to males [3]. The main clinical presentation involves repeated and symmetrical episodes of polyarthritis affecting several joints such as the hands, wrists, feet, and knees. Typical symptoms at the first stages include redness, swelling, fever, discomfort, and joint dysfunction [4]. The pathophysiology of RA is sophisticated and complex, and it is widely considered that several variables, including genetic and environmental factors, synergistically disrupt the immune system, resulting to an aberrant immunological response. Moreover, immune and non-immune cells work together to sustain chronic inflammation, resulting in decreased activity and eventually diminished quality of life [5]. In the initial phase of the disease, the immune system becomes active, leading to the infiltration of various inflammatory cells such as mast cells, macrophages, monocytes, fibroblasts, and chondrocyte-like cells into the synovial tissues of the affected joints. As the infiltration rises, RA gets progressively more severe and the synovial tissue is severely infiltrated by leukocytes, which finally leads to proliferation of the endothelium layer [6, 7]. Patients typically show increased levels of rheumatoid factor (RF), anti-citrullinated protein/peptide antibodies (ACPA), and other distinctive indicators [8].New pathways in the pathogenesis of RA include dendritic cell-T cell interaction, pyroptosis, and autophagy, which have been prominent in recent years [9, 10]. The primary therapeutic goals for RA involve pain relief, inflammation control, immune response suppression, cartilage protection, and disease progression delay [11]. Commonly prescribed medications include nonsteroidal anti-inflammatory drugs (NSAIDs), glucocorticoids, and disease-modifying antirheumatic drugs (DMARDs). Despite the availability of various treatment options, a significant number of RA patients do not achieve remission [12]. Signal transduction mechanisms are crucial in the development of RA, with abnormal activation of signaling pathways leading to the production of inflammatory molecules [13, 14]. As a result, identifying effective molecular targets for RA treatment is a key focus of research.

*PI3K* pathway governs fundamental processes such as cell survival, migration, proliferation, cytoskeletal remodeling, and immune cell homeostasis [15]. *PTEN*, an antagonistic phosphatase of various PI3Ks, ranks as the second most commonly mutated gene in human cancers after p53, indicating prognostic assessment in numerous tumors [16]. Beyond tumors, *PTEN* plays a pivotal role in various chronic inflammatory diseases, including chronic nephritis, gastritis, and RA. FLS constitute a significant cell population within the invasive pannus and function as crucial mediators of the local inflammatory response in RA [17]. Recent findings suggest that *PTEN* deficiency can amplify the survival and growth of FLS in RA, potentially due to *PTEN*’s regulatory role in the secretion and release of inflammatory factors associated with RA-FLS, including interleukin (*IL*)-*6/1β*, chemokine (C-C motif) ligand (*CCL*)-*23*, and vascular endothelial growth factor (*VEGF*) [18, 19]. Preliminary studies have explored the connection between PTEN and RA in the literature, however the precise processes responsible for the abnormal expression of PTEN in RA and its association with RA inflammation and FLS activation are not yet fully understood. We will review recent literature on the molecular structure of PTEN and its regulatory mechanisms. We will then discuss the evidence supporting PTEN as a potential target in RA, highlighting its involvement in regulating RA-FLS inflammation, joint damage in RA, and angiogenesis in RA. The goal is to thoroughly assess the existing knowledge and pinpoint areas where comprehension is lacking.

## Structure of PTEN

**Fig. 1:**
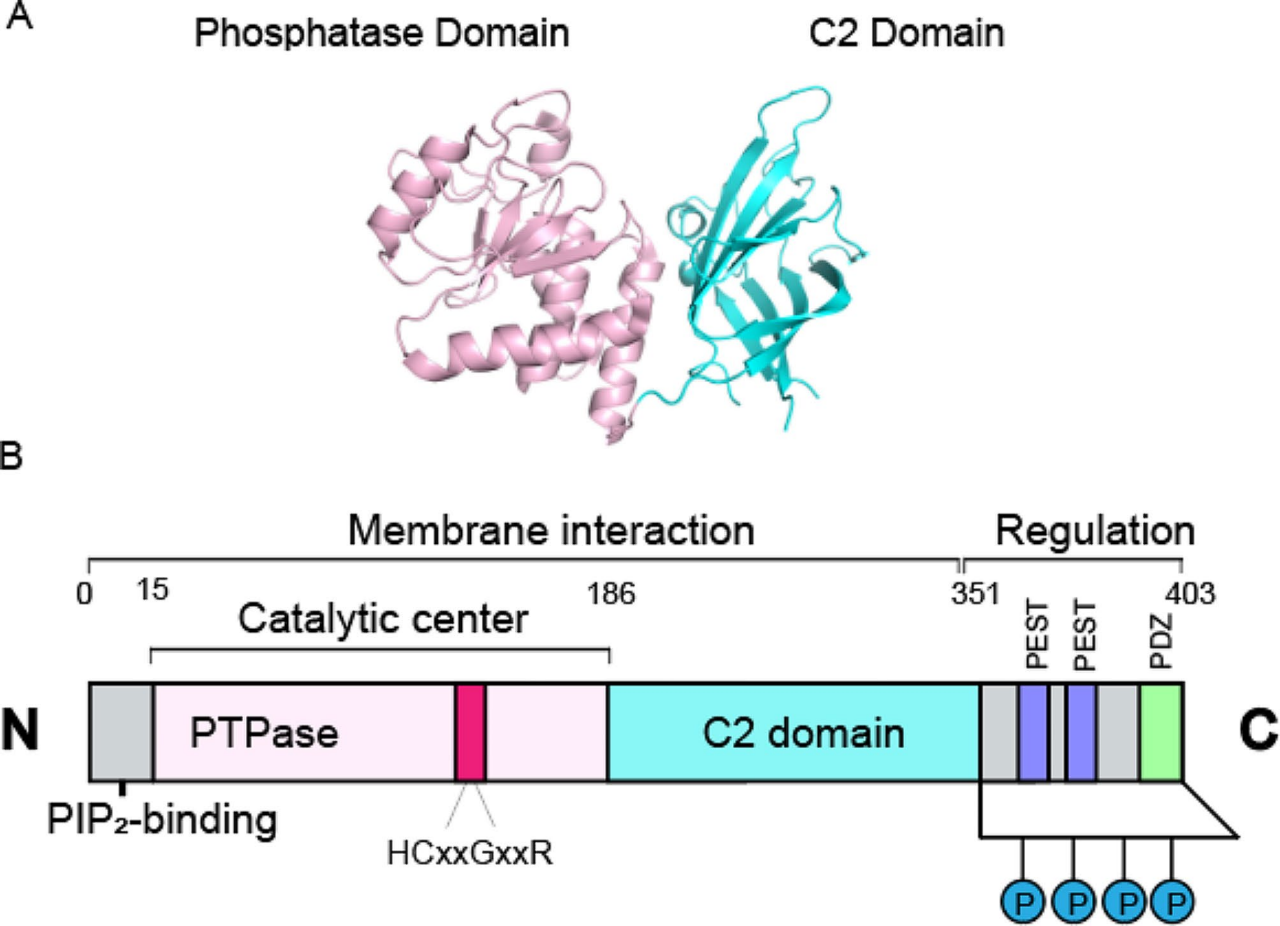
PTEN structures. **A**: Overall view of the PTEN crystal structure. The cartoon representation of the PTEN crystal structure (PDB ID: 7jvx) displays the phosphatase domain in pink and the C2 structural domain in blue-green. **B**: The domain architecture of PTEN. PTEN consists of 403 amino acids, and the protein has three functional domains: the N-terminal domain which binds and phosphatizes PI(4,5)P2, the C2 domain, and the carboxy-terminal tail domain (C-tail). The catalytically active site on the P-loop at the bottom of the active site pocket: HCxxGxxR (amino acids 123–130, red). The C2 domain plays a key role in membrane binding. PTEN also contains two PEST structural domains and a PDZ structural domain, PDZ binding domains can localize PTEN to specific cellular regions, and PEST sequences may be correlated with PTEN expression. there is a set of phosphorylation sites at the C-terminus that play an important role in maintaining PTEN stability and catalytic activity

*PTEN* is made up of 403 amino acids and has three functional domains (Fig. 1): the N-terminal [PI(4,5)P2]-binding/phosphatase domain, C2 domain, and carboxyl-terminal tail (C-tail) domain. The N-terminal domain of PTEN, namely residues 15–186, serves as the catalytic core, containing the HCxxGxxR active site within the P-loop (amino acids 123–130). Cancer-related missense mutations tend to accumulate in this specific area [20, 21]. The N-terminal phosphatase and C-terminal C2 structural domains play crucial roles in *PTEN*’s catalytic activity and intracellular distribution. The C-terminal C2 domain has structural features that enable attachment to the phospholipid bilayer, even though it does not have the loop structure for Ca^2+^ binding and Ca^2+^-dependent membrane contact [22, 23]. Additionally, N-terminal residues 6 to 15 participate in membrane interactions, particularly with the phospholipid PIP2, underscoring their importance in *PTEN*’s cellular function of down-regulating *PI3K* signaling [24]. The C-terminal region of *PTEN* contains identifiable motifs such as potential phosphorylation sites, a PDZ-binding motif, and two PEST motifs. Phosphorylating or dephosphorylating the serine and threonine residues at the C-terminal is crucial for maintaining the stability, positioning, and enzymatic function of *PTEN* [25, 26]. The PDZ-binding domain mostly interacts with particular cell surface receptors or intracellular proteins, aiding in *PTEN*’s localization to specific cellular areas [27, 28]. The PEST sequence functions as an intracellular marker for the half-life of proteins undergoing proteolytic degradation. Deletion of this region typically leads to an increase in *PTEN*’s expression [29]. The segment is not required for ubiquitin-ligase NEDD4-1 to ubiquitinate *PTEN* [30]. Further investigation is required to determine the roles of these two PEST sequences.

*PTEN* was first identified as a suppressor of the *PI3K* pathway in the cytoplasm, but it has also been found in the nucleus, where it can act as a tumor suppressor by regulating the cell cycle and maintaining genome stability, separate from its role in the *PI3K* pathway [31]. The labeling of cytoplasmic proteins into the nucleus is called nuclear localization signal (NLS), and most of the initially reported NLSs are abundant in basic amino acids, hence this form of NLS is named basic NLS or classical NLS (cNLS) [32]. These are classified into two distinct types: monopartite and bipartite. The former is a simple sequence consisting of 4–8 residues, like the first identified SV40 big T antigen NLS (PKKKRKV); the latter is two basic sequences with a separation of approximately 10–12 residues [33]. Various methods of *PTEN* transportation into the nucleus have been identified. Initially, *PTEN* moves through nuclear pore complexes (NPCs) to reach the nucleus [34]; Subsequently, the transport receptor Importin-11(*IPO11*) transfers PTEN to the nucleus and inhibits its breakdown in the cytoplasm. Importin-β (*IPO1*) and Transportin-2 (*TNPO2* or *IPO3*) are two proteins that facilitate transport [35, 36]. Additionally, oxidative stress hinders the nuclear export of *PTEN* to safeguard cells and control tumorigenesis. The inhibitory impact was determined to be associated with the phosphorylation of the amino acid residue Ser380 [37].

Notably, *PTEN* mRNA can undergo alternative translation initiation to generate multiple long isoforms of *PTEN* with additional N-terminal extensions. The initial ones identified were *PTEN*α (576 amino acids)and *PTEN*β (549 amino acids) [38]. *PTEN*α utilizes the same mRNA as *PTEN*, but it initiates translation from the non-AUG start codon CUG and includes an ATR region at the N-terminus [39]. *PTEN*α is a membrane-permeable lipid phosphatase that is secreted by cells and can enter other cells to function as a secreted *PI3K* antagonist. *PTEN*α is situated on the outer mitochondrial membrane and enhances mitochondrial autophagy by aiding in the recruitment of Parkinson disease protein 2(*PARK2*) to impaired mitochondria [40]. Moreover, research discovered that transcription activator-like effector nucleases (TALENs) -induced removal of PTENα in somatic cells hinders the operation of the mitochondrial respiratory chain. The translation of *PTEN*β is reported to be in-frame with the AUG start sequence of a conventional *PTEN*. *PTEN*β is mostly found in the nucleolus, where it directly interacts with and removes phosphate groups from nuciferin. Disruption of *PTEN*β results in an abnormal rise in pre-rRNA production and encourages cell growth [41]. Aside from *PTEN*α and *PTEN*β, there are a minimum of four other N-terminally elongated versions of *PTEN* that can be produced by utilizing the start codon located in the 5′UTR region of the *PTEN* mRNA [42]. Recent investigations have shown the existence of *PTEN*ε (475 amino acids), whose natural reduction leads to the development of filamentous pseudopods and boosts the spreading ability of cancer cells [42].

## PTEN functions

**Fig. 2:**
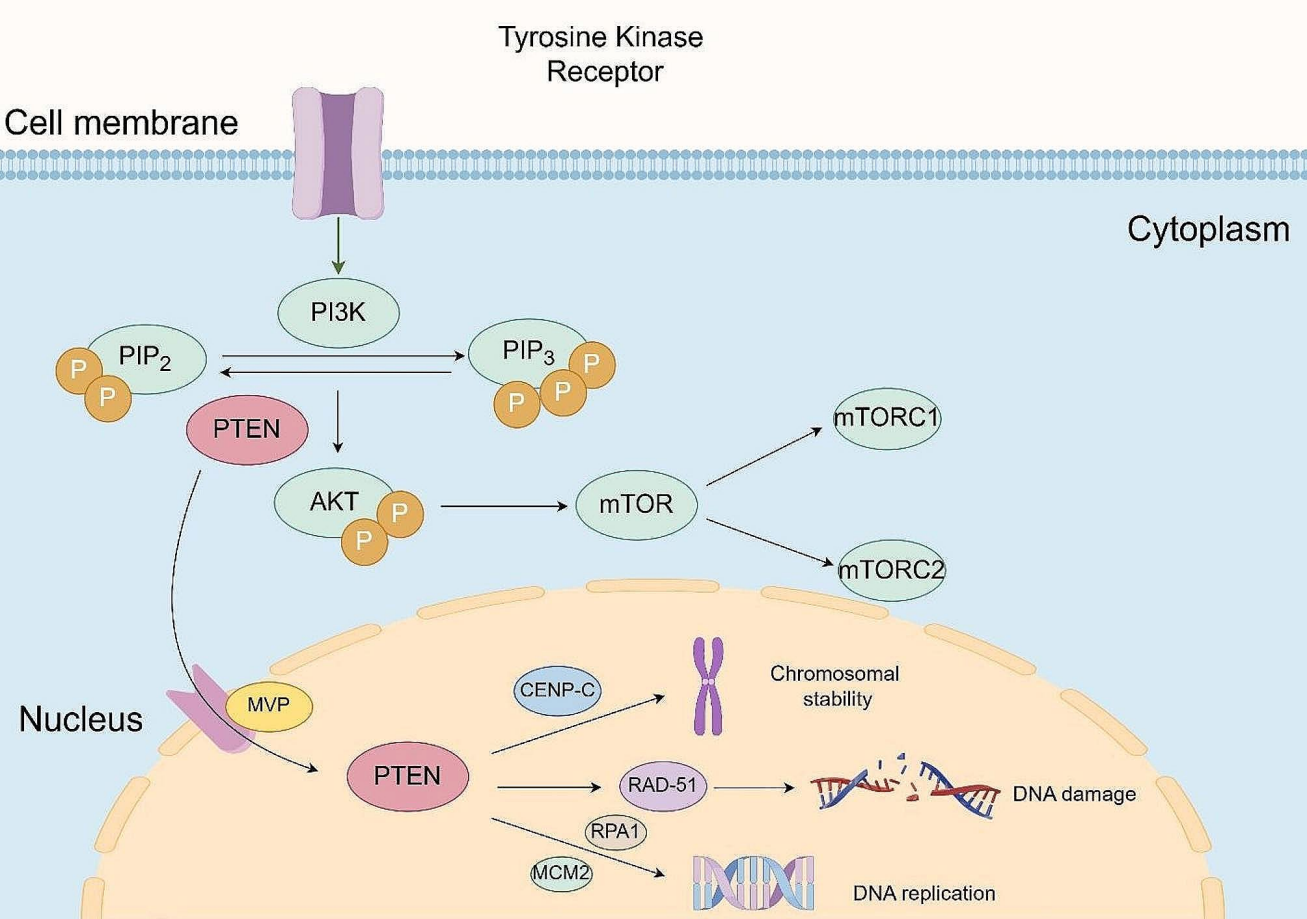
Illustrates the functions of PTEN in the cytoplasm and nucleus. **A**: Persistent activation of the PI3K/AKT signaling pathway and subsequent mTOR activation upon PTEN loss. This cascade, initiated by the phosphorylation of downstream effector proteins, contributes to processes such as protein and nucleotide synthesis. **B**: PTEN interacts with MVP in the nucleus in a Ca + dependent way and has a significant function in preserving genome stability while interacting with various proteins

*PTEN* stands as the inaugural tumor suppressor gene identified to possess dual phosphatase characteristics for both lipid and protein phosphatases [43]. Operating within the cytoplasm and cell membrane, PTEN governs pivotal cellular processes, including cell survival, proliferation, senescence, and angiogenesis (Fig. 2). This regulation occurs predominantly through inhibiting the PI3K-AKT pathway, primarily executed via lipophosphatase activity [44, 45]. Upon reception of external signals, such as growth factors or insulin, cells activate *PI3K*, leading to the generation of PIP3. PIP3, in turn, binds to *AKT*, initiating *AKT* phosphorylation expression and impeding apoptotic signaling pathways, thereby fostering cell survival [46]. Furthermore, *AKT* can indirectly stimulate proteins associated with the mechanistic target of rapamycin (*mTOR*). The activation of *mTOR* assumes a pivotal role in diverse cellular activities, encompassing protein synthesis, autophagy, nucleotide synthesis, and cell growth, achieved through the phosphorylation of various downstream effector proteins [43]. *PTEN*, functioning as a critical pathway regulator, effectively thwarts *AKT* activation by dephosphorylating PIP3 and converting it to PIP2. Consequently, this process sustains in vivo homeostasis [47].

*PTEN* is primarily known as a lipid phosphatase that hinders the activation of the oncogenic *PI3K-AKT* signaling pathway. *PTEN* has additional potential modes of action, such as protein phosphatase activity. Qi et al. showed that the protein phosphatase function of *PTEN* is necessary for epithelial differentiation and polarization in the epiblast [48]. Furthermore, *PTEN* controls cell cycle and death by utilizing its protein phosphatase activity, performing a similar function to *AKT* [49]. Xu et al. discovered that *PTEN*’s protein phosphatase activity dephosphorylates and inhibits autophosphorylated phosphoglycerate kinase 1 (*PGK1*), leading to the inhibition of glycolysis, ATP synthesis, and brain tumor cell proliferation [50]. In addition, Helen and colleagues utilized the mutant enzyme *PTEN*^Y138L^, which specifically lacks protein phosphatase activity, to study *PTEN* in transgenic mice lacking either all *PTEN* function or only protein phosphatase activity in the prostate gland. They discovered that *PTEN* protein phosphatase activity was not essential for tumor suppression [51]. *PTEN*α deficiency enhances mitochondrial autophagy triggered by different mitochondrial-damaging substances. A laboratory test showed that *PTEN*α removes phosphate groups from pSer65-Ub using its protein phosphatase activity, regardless of its lipid phosphatase function [39]. *PTEN* plays crucial roles in the nucleus by regulating chromosome integrity, chromatin structure, DNA replication, and damage repair [52]. *PTEN* gains access into the nucleus through a calcium-dependent pathway by interacting with the main vault protein (*MVP*) [34]. Subsequently, genome stability is maintained via its interaction with CENP-C, a centromere-specific binding protein, which is essential for ensuring centromere stability. *PTEN* also has a role in regulating the expression of RAD51, a key protein involved in repairing DNA damage from double-stranded breaks (DSBs) [53, 54]. Moreover, *PTEN* is crucial in DNA replication since it interacts with many replication-related proteins such as replication protein A1 (*RPA1*) and microchromosome maintenance complex component 2 (*MCM2*) at DNA replication forks [55]. A study revealed that the activation of the G2/M checkpoint, triggered by the DNA damage response (DDR), involves the engagement of *p53* and *PTEN* in NSCLC cells using a dynamic Boolean network and experimental data [56].

## Mechanisms of PTEN activity regulation

The loss of *PTEN* function typically results in the accumulation of PIP3, subsequently activating the *AKT* signaling pathway. Additionally, *PTEN* deficiency is associated with the activation of multiple pathways through PIP3-dependent signaling, including the *Ras-MAPK*, *Wnt/β-catenin*, *Notch*, and *Hippo* pathways [43]. Predominant efforts to reinstate *PTEN* loss have predominantly focused on inhibiting *PI3K*. However, a substantial proportion of disorders, encompassing tumors, exhibit impairment in *PTEN* activation [57]. As depicted in Table 1, various regulatory mechanisms influence *PTEN* activity, including transcriptional regulation (involving miRNAs) and post-transcriptional translational modifications such as phosphorylation, ubiquitination, acetylation, and methylation [58].

**Table 1:** The regulatory mechanism of PTEN activity

Regulation	Suggested mechanism	Specific examples	References
Transcriptional regulation	protein-protein interaction	RBM24 directly binds and stabilizes PTEN mRNA Silencing of YTHDC1 decreases PTEN expression and destabilizes PTEN mRNAs	[59] [60]
	IncRNA	lncRNA UFC1 binds to EZH2 and mediates its aggregation in the promoter region of the PTEN gene, epigenetically silencing PTEN expression	[61]
	miRNA targeting	miR-26a-5p targeting the 5′UTR of PTEN promotes cell proliferation and G1/S transition in RA-FLS	[64]
		miR-214 activates the AKT signaling pathway by directly targeting PTEN and MIR31HG	[65]
		miR-3-1p promotes IL-1β-induced chondrocyte pyroptosis by inhibiting PTEN expression	[66]
		miR-10b promotes RA progression by targeting PTEN to disrupt the balance between CD4 T cell subsets	[67]
Post-translational modifier	ubiquitination	HRD1, STX3, and GFPT1 promote ubiquitination degradation of PTEN, which promotes cancer cell proliferation in vivo	[69–71]
		The ubiquitination of PTEN at lysine position 13 regulates its nuclear localization and suppresses DNA damage in vivo	[73]
		Ubiquitination at lysine 221 leads to degradation of PTEN and indirectly impairs HR leading to instability of DSB	[74]
	phosphorylation	The enhancement of DNA repair processes, mediating resistance to DNA damage, is facilitated by the phosphorylation of PTEN at tyrosine 240	[75]
		Phosphorylation of PTEN serine 113 associated with PTEN nuclear translocation and autophagy	[76]
		The proper activation of the S-phase checkpoint, regulated by PI3K-p27, is contingent upon the phosphorylation of PTEN at position 398	[77]
	acetylation	The interaction of PTEN with proteins containing PDZ structural domains can potentially be regulated by the acetylation of PTEN at the lysine 402 position	[78]
	methylation	PTEN methylation promotes FLS inflammation and activation	[83]
		PTEN hypermethylation promotes angiogenesis in experimental arthritis	[84]

### Transcriptional regulation

Regulating *PTEN* levels includes several methods. RNA-binding motif protein 24 (*RBM24*) binds directly to *PTEN* mRNA during the transcription step, thereby stabilizing it [59]. m^6^A reader *YTHDC1* silencing decreases *PTEN* expression and triggers the *PI3K/AKT* signaling cascade by destabilizing *PTEN* mRNA in a m^6^A-dependent mechanism [60]. An exosome-transmitted long non-coding RNA (lncRNA) called *UFC1* binds to Enhancer of Zeste Homolog 2 (*EZH2*) and assists in its clustering in the promoter area of Phosphatase and *PTEN*, leading to the epigenetic suppression of *PTEN* expression [61]. MicroRNAs (miRNAs) are short non-coding RNAs, typically 19–25 bp in length, that regulate physiological activities by targeting messenger RNA molecules. Their importance covers both normal and abnormal functions, leading to thorough research on different diseases to understand the molecular and signaling mechanisms involved in disease development. miRNAs have a significant role in illness development, making them promising candidates for biomarkers and therapeutic targets [62]. Therefore, this review will focus on miRNA targeting and regulation for the aim of summarization. Several miRNAs, such as miR-223-3p, miR-486-3p, and miR-23a-3, have been found to be abnormal in RA-FLS. The miRNAs show varying levels of expression. An enrichment study of miRNA targets shows that the discovered target genes, such as signal transducer and activator of transcription 3 (*STAT3*), PR domain zinc finger protein 1 (*PRDM1*), and *PTEN*, are enriched in crucial processes associated with B cell activation, differentiation, and receptor signaling [63].

Multiple studies suggest that *PTEN* could be a target gene for miRNA-mediated regulation in RA-FLS. Increased levels of miR-26a-5p in RA-FLS lead to enhanced cell proliferation, G1/S transition, cell invasion, and anti-apoptotic effects. Conversely, reducing miR-26a-5p has the opposite effect. The effects are presumably caused by the activation of the *PI3K/AKT* signaling pathway by miR-26a-5p, which occurs by targeting the 5′UTR of *PTEN*. Therefore, miR-26a-5p is a suitable clinical target for developing innovative therapeutic methods for RA [64]. The work further shows that *miR-214* promotes the *AKT* signaling pathway by directly targeting *PTEN* and *MIR31HG*. Disrupting the *MIR31HG*-*miR-214*-*PTEN* pathway inhibits the growth, movement, and production of inflammatory factors and MMPs in RA-FLS [65]. miR-3-1p, activated by specificity protein 1(*SP1*), enhances chondrocyte pyroptosis triggered by *IL-1β* by suppressing *PTEN* and activating the *PINK1/Parkin* pathway [66]. This discovery offers new perspectives on investigating miRNA-induced localized cell death and the possible pathways involved in the development of rheumatoid arthritis. MiR-10b disturbs the equilibrium of CD4 T cell subsets, leading to the progression of rheumatoid arthritis. *PTEN* was discovered as a target of miR-10b. Using *PTEN* siRNA increased Th17 cells and decreased Treg cells [67]. When the *AKT/mTOR* pathway is inhibited, miRNAs significantly downregulate the expression of phosphatase and *PTEN* [68].

### Post-translational modifier

*PTEN* activity is regulated through several changes, including ubiquitination, phosphorylation, acetylation, and methylation. Here, we provide a summary of recent discoveries in this field. HMG-CoA reductase degradation protein 1(*HRD1*), Syntaxin3(*STX3*), and Glutamine–Fructose-6-Phosphate Transaminase 1(*GFPT1*) have been recognized as facilitators of *PTEN* degradation through ubiquitination, resulting in enhanced cancer cell proliferation [69–71]. Nuclear *PTEN* is more likely to be deleted than cytoplasmic *PTEN*, as it is less stable and more prone to degradation through the ubiquitin-proteasome pathway [72]. Lysine 13 ubiquitination controls the nuclear localization of *PTEN*, preventing DNA damage in living organisms [73]. *PTEN* degradation occurs due to the ubiquitination of lysine 221, leading to a decrease in *RAD51* expression and causing instability in double-strand breaks (DSB) by hindering homologous recombination (HR) [74]. Phosphorylation of *PTEN* at tyrosine 240 boosts DNA repair mechanisms and provides protection against DNA damage. Crucially, the distinct phosphorylation action of *PTEN* functions independently of its lipid phosphatase activity [75]. ATM phosphorylates *PTEN* at serine 113, promoting the movement of *PTEN* into the nucleus and triggering autophagy [76]. The activation of the S-phase checkpoint is dependent on the phosphorylation of *PTEN* at position 398, which is regulated by *PI3K-p27* [77]. Acetylation of lysine 402 on *PTEN* may regulate its interaction with proteins that have the PDZ structural domain [78]. Deacetylating *PTEN* hinders the ubiquitination of epidermal growth factor receptor (*EGFR*), which strengthens extracellular matrix formation, triggers autophagy in chondrocytes, and offers defense against osteoarthritis [79].

DNA methylation is a crucial epigenetic alteration in animals that has important impacts on the growth, development, and progression of diseases, particularly in cancer research. Changes in DNA methylation in RA-FLS occur early in the progression of RA, before a confirmed clinical diagnosis is made. DNA methylation is suggested as a possible main cause of RA persistence [80]. A study found a link between the immunological response in the blood of people with active RA and changes in DNA methylation patterns of circulating monocytes. The study found many clusters of CpG sites with methylation levels closely linked to the disease activity ratings of individuals [81]. Recent research has revealed a significant link between tumors and autoimmune illnesses, emphasizing hypermethylation as a route for *PTEN* inactivation. Li and colleagues suggested that the decrease in *PTEN* expression may be associated with CpG site methylation. This was supported by the discovery of four CpG sites near and before the first exon of the transcript that had a high concentration of C and G nucleotides. The methylation inhibitor 5-Azacytidine significantly increased *PTEN* expression in adjuvant-induced arthritis (AIA) and reduced the mRNA and protein levels of Tumor necrosis factor alpha (*TNF-α*), Interleukin 6 (*IL-6*), and *IL-1β* [82]. Recent research highlights the influence of *PTEN* methylation on inflammation and activation in FLS. Preventing *PTEN* methylation led to increased amounts of anti-inflammatory molecules and decreased activation of FLS in people with RA [83]. DNMTs catalyze the onset of DNA methylation, and their inhibition in experimental arthritis angiogenesis suppresses DNMT1-mediated *PTEN* hypermethylation [84].

## PTEN as an emerging target in RA

### Involved in RA-FLS inflammatory regulation

**Fig. 3:**
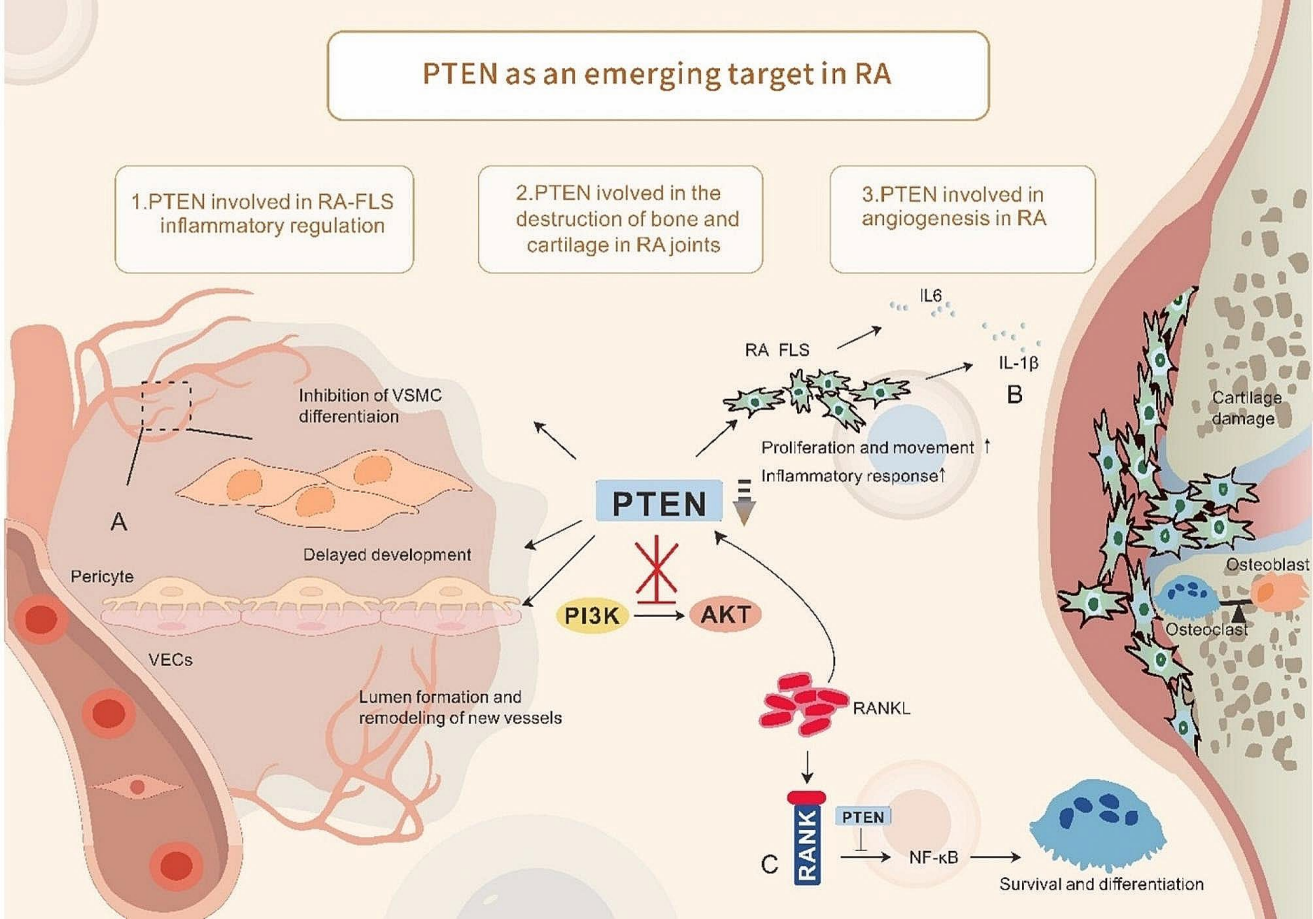
Description of PTEN as an emerging target in RA. **A**: PTEN deficiency promotes VECs movement and infiltration; delayed pericyte development; and inhibition of VSMCs differentiation, ultimately leading to an imbalance in vascular homeostasis. **B**: PTEN deficiency promotes the proliferation and invasion of FLS and the formation of a proactive inflammatory relief, leading to excessive synovial tissue formation and ultimately joint injury. **C**: PTEN deficiency disrupts the balance between osteoblasts and osteoclasts, promotes osteoclast survival, decreases chondrocyte viability, and accelerates bone degeneration

RA-FLS, specialized mesenchymal cells, are found in the synovium of both joints. RA causes FLS to display many biological characteristics such as aberrant growth, increased aggressiveness, and excessive secretion of inflammatory substances and cytokines, including *TNF-α*, *IL-6*, C-C chemokine ligand(*CCL-2*), Matrix metalloproteinase-3(*MMP3*), and vascular endothelial growth factor-alpha (*VEGF-α*). These processes lead to excessive synovial tissue formation and eventual joint damage [85, 86]. The molecular pathways responsible for activated FLS proliferation and migration are not well understood, however *PTEN* may play a role in this complex pathway [19]. Studies show that the absence of *PTEN* results in the creation of substances that cause arthritis and degeneration of the joints [87]. Increasing *PTEN* expression leads to alterations in the *PI3K/AKT* signaling pathway, resulting in a delay in RA progression [88]. Reducing *PTEN* expression using either the *PTEN* inhibitor BPV or PTEN-RNAi was found to increase the growth and movement of FLS [19] (Fig. 3B). NFIC, a part of the NFI family, acts as a transcription factor that boosts *PTEN* transcription. This process results in RA-FLS cell death and successfully decreases inflammation in mice with collagen-induced arthritis (CIA) [89]. Glutathione (GSH), an antioxidant, reduces inflammation in FLS by adjusting pathways associated with *PTEN* [90]. Boosting *PTEN* levels in inflamed tissues using recombinant adenoviruses or decreasing *PTEN* phosphorylation markedly reduced the inflammatory reaction by inhibiting *AKT* [88, 91]. *PTEN* overexpression reduced T-cell activation and influenced the development of Th17 and Treg cells, improving experimental autoimmune arthritis. The progression of autoimmune arthritis is hindered by the inadequate levels of *PTEN* in myeloid cells, which limits the development of harmful Th17-type immune responses [22, 92]. The *PI3K/PTEN* pathway is suggested to regulate various aspects of disease development in inflammatory conditions in different tissues, including cell movement, invasiveness, cytokine generation, cell growth, and T-cell orientation. The results enhance comprehension of regulatory complexities, as seen in Table 2.

**Table 2:** The mechanism of PTEN regulating RA

Relationship between PTEN and RA	Effects of PTEN loss in RA	References
Modulation of RA-FLS inflammation	Promotes the formation of arthritic aggressors and joint degeneration Promote proliferation and migration of FLS Promoting PTEN expression or reprogramming PTEN to induce RA-FLS, apoptosis and alleviate FLS inflammation Blocking pathogenic Th17-type immune responses	[87] [19] [86] [22]
Involved in the destruction of bone and cartilage	Indirectly involved in the regulation of angiogenic and osteoclastogenic factors, providing potential signals for the shaping of the inflammatory microenvironment Elevated osteoclastogenic capacity of myeloid cells contributes to intensified localized bone destruction associated with increased inflammation	[99] [100]
	Indirectly induced osteoclasts enhanced NFATc1 expression, leading to a significant increase in terminal differentiation of osteoclasts in vitro	[101]
	Faster osteoblast differentiation and greatly reduced apoptosis in vitro Association with miRNAs involved in the regulation of mature osteoclast survival Promoting mitochondrial autophagy in osteoclast precursors thereby inhibiting osteoclast formation Decreased chondrocyte viability and type II collagen production	[104] [105] [106] [107]
Involved in angiogenesis	Enhanced proliferation, migration, invasion and angiogenesis of VECs The biological function of HUVECs in inhibiting angiogenesis is augmented by the overexpression of PTEN PTEN overexpression reduces angiotensin II-induced damage and decreases fibrosis and inflammatory markers Promotes maintenance of smooth muscle cell differentiation and reduces pathological vascular remodeling Facilitated injury-induced vascular adaptation Generalized upregulation of inflammatory and fibrosis-related genes The establishment of an inflammatory phenotype is marked by the recruitment of progenitor cells derived from the bone marrow	[112] [113] [115] [116] [117] [118] [119]

### Involved in the destruction of bone and cartilage in RA joints

RA is defined by inflammation that results in the deterioration of bone and cartilage in the joints it affects. RA triggers an inflammatory response that leads to bone loss by increasing osteoclastic bone resorption and reducing osteoblastic bone growth [93, 94]. This imbalance results in bone degradation, loss of bone around the joints, and widespread osteoporosis. Osteoclasts play a crucial role in bone degradation as shown in basic research and the efficacy of antibody treatments produced by osteoclasts in medical practice [95]. Osteoclasts originate from hematopoietic stem cells and differentiate within the monocyte-macrophage lineage from osteoclast precursors [96]. Recent research indicates that *PI3K* has a role in osteoclast formation, and activating the *PI3K/AKT/mTOR* pathway can enhance osteoblast development. Specific *PI3K/AKT* inhibitors, including LY294002 and LY3023414, have shown the capacity to decrease bone growth in both living organisms and laboratory settings [97, 98]. *PTEN* loss has been linked to increased Early growth response factor 1 (*EGR1*) expression, which plays a role in controlling osteoclast formation and encouraging metastasis [99]. Friedrich’s research suggests that removing *PTEN* and maintaining the lack of *PI3K* signaling in myeloid cells can enhance osteoclastogenesis in myeloid cells, leading to localized bone degradation [100]. Liu et al. used Cre-mediated recombination to selectively disrupt the *PTEN* in osteoblasts. Osteoblasts lacking *PTEN* developed faster, had lower apoptosis rates, and showed a notable rise in phosphorylated *AKT* levels compared to the control samples [101].

Two vital cytokines, macrophage colony-stimulating factor (M-CSF) and receptor activator of nuclear factor-kappa B ligand (RANKL), are essential for the differentiation of osteoclasts [102, 103]. RANKL, a cytokine attached to the *TNF* superfamily and cell membrane, interacts with receptor activator of nuclear factor-kappa B (RANK)on osteoclast precursors, triggering osteoclast development by activating nuclear factor of activated T-cells 1 (*NFATc1*) as a key regulator. *RANKL* affects *PTEN* in two ways in the control of bone homeostasis (Fig. 3B). Initially, it suppresses the expression and function of *PTEN* phosphatase, promoting cell survival and growth. Concurrently, RANKL stimulation triggers the *PI3K/AKT* cascade, resulting in the deactivation of *PTEN* and Glycogen synthase kinase 3 Beta (*GSK3β*), ultimately facilitating osteoclast formation [104]. *PTEN* loss boosts the final development of RANKL-induced osteoclasts via elevating *NFATc1* expression, as demonstrated in in vitro experiments [105]. *PTEN* has been discovered to interact with miRNAs that control the survival of mature osteoblasts. Enhancing mitochondrial autophagy in osteoclast precursors hinders osteoclast development and decreases bone damage in collagen-induced arthritic mice. The PINK1/Parkin pathway mainly controls mitochondrial autophagy [106]. Notably, *PTEN* has two distinct roles. Its overexpression in degenerating disc cells leads to apoptosis in these cells, reducing chondrocyte viability and inhibiting the formation of type II collagen by blocking *PI3K/AKT* activation [107].

### Involved in angiogenesis in RA

Angiogenesis is crucial for promoting effective bone regeneration by allowing the transfer of nutrients, growth factors, and waste products, which helps maintain the internal balance of the skeleton [108]. Excessive angiogenic factors in RA counteract anti-angiogenic factors, resulting in increased endothelial cell infiltration, heightened synovial inflammation, and eventual bone and cartilage destruction. Inhibiting angiogenesis in the joint has the potential to reduce synovitis and limit the growth of sub-synovial tissue [86, 109]. Targeting angiogenesis could be a crucial strategy in treating RA [110]. Various pro-inflammatory mediators involved in stimulating the formation of new blood vessels in rheumatoid arthritis are being assessed as possible targets for upcoming treatments. These consist of chemokines (*CXCL12*), cytokines (*IL-17*, *IL-18*, and *MIF*), growth factors (*Ang1* and *Ang2*), proteases (*MMPs*), and adhesion molecules (*ICAM1* and *VCAM1*) [111]. Approved treatments for rheumatoid arthritis that may work by inhibiting angiogenesis include *TNF*, *IL-1β*, and *IL-6* inhibitors, thalidomide, and Cox-2 inhibitors.

*PTEN*’s involvement in RA angiogenesis is becoming more acknowledged f(Fig. 3A). Suppressing *PTEN* expression has been demonstrated to enhance the growth, movement, infiltration, and formation of new blood vessels by vascular endothelial cells (VECs) [112]. Increased PTEN expression boosts the biological activities of HUVECs, resulting in a suppressive impact on angiogenesis [113]. Intravascular homeostasis is maintained through the cooperation of endothelial cells, pericytes, and vascular smooth muscle cells (vSMCs), which play a crucial role in stabilizing and controlling vascular function. Pericyte deficiency is linked to several disorders such as diabetic retinopathy and cancer [114]. *PTEN* loss causes a delay in pericyte development, leading to the activation of *PI3K* signaling. Pericyte maturation is necessary for vascular remodeling during angiogenesis [13]. Increased expression of *PTEN* reduces angiotensin II-induced damage while also decreasing fibrosis and inflammatory indicators [115]. 5-Azacytidine, a DNMT1 inhibitor, has been found to enhance *PTEN* expression, promote the preservation of smooth muscle cell differentiation, and reduce pathological vascular remodeling [116]. Depletion of *PTEN* in mice leads to prolonged *PI3K-AKT-mTOR* signaling, resulting in decreased expression of vSMC markers. The decrease occurs simultaneously with the activation of *NF-κβ* and the production of chemokines and substances that promote fibrosis (*MCP-1*, *IL-6*, and *KC/CXCL1*), aiding in injury-induced vascular adjustment [117]. Interestingly, *PTEN* loss was associated with neointima development, leading to the increased expression of genes associated with inflammation and fibrosis [118]. Stromal cell-derived factor-1 alpha (*SDF-1α*) acts as an intermediary following *PTEN*. When *PTEN* is lost in vascular smooth muscle cells, it causes an upregulation of *SDF-1α* expression, leading to the emergence of an inflammatory phenotype characterized by the attraction of bone marrow-derived progenitor cells [119]. Moreover, important targets further along the *PTEN* pathway have been associated with abnormal vascular adaptation. The findings highlight the significant role of *PTEN* signaling in rheumatoid arthritis angiogenesis, involving endothelial cells and all components of the arterial wall structure [120].

## Discussion and outlook

RA is a prevalent autoimmune inflammatory condition. If left untreated, persistent inflammation of the joint lining can result in significant joint deterioration, disability, and inability to function [121]. *PTEN* is the initial oncogene identified to possess bispecific phosphatase activity, and is a gene that is highly linked to cancer following the *p53*. The quantity of articles on *PTEN* is growing annually. *PTEN* in the cytoplasm is known to operate as a negative regulator of the *PI3K* pathway, influencing the RA inflammatory response, bone degradation, and angiogenesis. The decrease of *PTEN* activity occurs through various routes. At the transcriptional stage, *PTEN* is silenced by epigenetic mechanisms by the targeted control of numerous proteins. Recent research has shown that inhibiting the PIP3 phosphatase activity of *PTEN* can be achieved by targeting the *PTEN* catalytic core (aa118-141) [122]. *PTEN* activity is controlled through ubiquitination, phosphorylation, acetylation, and methylation changes in the translational phase. DNA methylation is being viewed as a potential target for therapeutic and diagnostic purposes in RA. The transcription products of *PTEN* contain numerous CpG sites, therefore, the main attention should be on the effects of *PTEN* hypermethylation on the disease. *PTEN* is present in the nucleus and has a role in regulating DNA damage, maintaining genomic stability, inhibiting oncogenic transcription, and is not dependent on the *PI3K* pathway. Deletion of nuclear *PTEN* is linked to many cancer characteristics. Post-translational changes are essential for the nuclear translocation and stability of *PTEN*. Ultimately, the cause of RA is intricate, and examining the *PTEN*’s upstream and downstream actions can provide valuable understanding of gene regulation’s complexity and diversity.

An urgent need exists in the clinical field for new antirheumatic drugs that show increased effectiveness [123]. Current research suggests that using *PTEN* as a therapeutic target for RA is still in the early stages. Illustrative methods involve using adenoviral or genetically modified bionic membrane-encapsulated vectors for mRNA therapies, employing herbal extracts to adjust the molecular mechanisms related to the anti-inflammatory or immunomodulatory effects of *PTEN*, and using methylation inhibitors or antioxidants to alter pathways associated with *PTEN* [88, 124, 125]. Among them, Traditional Chinese remedies provide beneficial therapeutic effects on RA, offering a greater number of targets and less adverse effects compared to conventional therapeutic pharmaceuticals. They are a key area of interest in RA drug development research. Guizhi Shaoyao Zhimu granules (GSZGs) enhance the process of autophagy in mitochondria of osteoclast precursors through the *PTEN*-induced *PINK1*/*Parkin* pathway, resulting in reduced bone degradation in mice with CIA [106]. Catalpol is a bioactive compound derived from the traditional herb Radix Rehmanniae. Catalpol increases *PTEN* function by reducing *PTEN* ubiquitination and degradation, which leads to the inhibition of the *RANKL*-induced *NF-κB* and *AKT* signaling pathways. It shows promise for treating RA and other bone-related disorders [126].Overall, Strategies for one-way control of *PTEN* expression levels should be further explored in future medication development. RA herbal formulae derived from traditional chinese medicine (TCM) clinical practice require additional testing in vivo and in vitro. It is essential to produce active components with reduced side effects and broader target coverage in collaboration with pharmacology. Future research should concentrate on investigating the role of *PTEN* in the progression of RA to enhance comprehension and offer direction for the diagnosis and treatment of RA.

## Data Availability

No datasets were generated or analysed during the current study.

## Abbreviations

PTEN: Phosphatase and tensin homolog deleted on chromosome 10
PI3K/AKT: phosphatidylinositol 3-kinase (PI3K)/protein kinase B
RA: rheumatoid arthritis
FLS: fibroblast-like synoviocytes
RF: rheumatoid factor
ACPA: anti-citrullinated protein/peptide antibodies
NSAIDs: nonsteroidal anti-inflammatory drugs
DMARDs: disease-modifying antirheumatic drugs
VEGF: vascular endothelial growth factor
C-tail: carboxyl-terminal tail
NLS: nuclear localization signal
cNLS: classical NLS
NPCs: nuclear pore complexes
IPO1: Importin-β
IPO11: Importin-11
TNPO2: Transportin-2
TALENs: transcription activator-like effector nucleases
PGK1: phosphoglycerate kinase 1
MVP: main vault protein
DSBs: double-stranded breaks
RPA1: replication protein A1
MCM2: maintenance complex component 2
DDR: DNA damage response
RBM24: RNA-binding motif protein 24
EZH2: Enhancer of Zeste Homolog 2
miRNAs: MicroRNAs
STAT3: signal transducer and activator of transcription 3
PRDM1: PR domain zinc finger protein 1
HRD1: HMG-CoA reductase degradation protein 1
STX3: Syntaxin3
GFPT1: Glutamine–Fructose-6-Phosphate Transaminase 1
DSB: double-strand breaks
HR: homologous recombination
EGFR: epidermal growth factor receptor
AIA: adjuvant-induced arthritis
TNF-α: Tumor necrosis factor alpha
IL-6: Interleukin 6
CCL-2: C-C chemokine ligand
MMP3: Matrix metalloproteinase-3
VEGF-α: vascular endothelial growth factor-alpha
CIA: collagen-induced arthritis
GSH: Glutathione
M-CSF: macrophage colony-stimulating factor
RANKL: receptor activator of nuclear factor-kappa B ligand
RANK: receptor activator of nuclear factor-kappaB
NFATc1: nuclear factor of activated T-cells 1
GSK3β: Glycogen synthase kinase 3 Beta
VECs: vascular endothelial cells
vSMCs: vascular smooth muscle cells
GSZGs: Guizhi Shaoyao Zhimu granules
TCM: Traditional Chinese Medicine
